# Thrombotic Complications in Patients with Immune-Mediated Hemolysis

**DOI:** 10.3390/jcm10081764

**Published:** 2021-04-18

**Authors:** Marco Capecchi, Alessandro Ciavarella, Andrea Artoni, Maria Abbattista, Ida Martinelli

**Affiliations:** 1 Department of Biomedical Sciences for Health, Università degli Studi di Milano, 20133 Milan, Italy; alessandro.ciavarella@unimi.it; 2 Angelo Bianchi Bonomi Hemophilia and Thrombosis Center, Fondazione IRCCS Ca’ Granda Ospedale Maggiore Policlinico, 20122 Milan, Italy; andrea.artoni@policlinico.mi.it (A.A.); maria.abba88@gmail.com (M.A.); ida.martinelli@policlinico.mi.it (I.M.)

**Keywords:** hemolytic anemia, paroxysmal nocturnal hemoglobinuria, thrombosis, anticoagulants, thrombophilia

## Abstract

Autoimmune hemolytic anemias are rare and heterogeneous disorders characterized by hemolysis, which is a well-recognized risk factor for thrombosis. The most common immune-mediated anemias are represented by autoimmune hemolytic anemia and paroxysmal nocturnal hemoglobinuria, both associated with a high rate of thrombosis. Multiple pathophysiological mechanisms for thrombosis have been proposed, involving hemolysis itself and additional effects of the immune system. Despite the increasing awareness of the thrombotic risk in these conditions, evidence-based guidance on prevention and management of thrombotic events is lacking. We herein report available evidence on epidemiological data on thrombosis and thrombophilia in immune-mediated hemolysis, together with possible underlying pathophysiological mechanisms. In addition, we summarize current recommendations for treatment of thrombosis in immune-mediated hemolysis. In particular, we address the issue of thrombotic complications treatment and prophylaxis by proposing a therapeutic algorithm, focusing on specific situations such as splenectomy and pregnancy.

## 1. Introduction

Hemolysis is a well-recognized risk factor for venous and arterial thrombosis, regardless the underlying cause leading to its onset. An increased risk of thrombosis is associated with various red blood cell hemolytic disorders, such as hemoglobinopathies, red blood cell membrane disorders and immune-mediated hemolytic anemias [1]. Multiple pathophysiological mechanisms have been proposed, including systemic inflammation, nitric oxide scavenging, von Willebrand factor alterations, all secondary to increased plasma levels of free hemoglobin, heme and iron, and the release of red blood cell-derived prothrombotic microvesicles [2]. Immune-mediated hemolysis, namely autoimmune hemolytic anemia (AIHA) and paroxysmal nocturnal hemoglobinuria (PNH), is characterized by a higher rate of thrombosis compared to the other aforementioned red blood cell hemolytic disorders [1], as a result of an additional effect of the immune system. Despite the increasing awareness of the thrombotic risk in immune-mediated hemolysis, evidence-based guidance on prevention and management of thrombotic events is lacking.

In this review, epidemiological data on the incidence rates of thrombosis in immune-mediated hemolysis and pro-thrombotic risk factors will be reported together with possible pathophysiological mechanisms leading to thrombosis. In addition, current clinical management will be reviewed and a diagnostic and therapeutic approach will be proposed, with a particular attention to specific situations such as splenectomy and pregnancy.

## 2. Incidence and Risk Factors for Venous or Arterial Thrombosis in Immune Mediated Hemolysis

AIHA is a group of rare (1–3 cases per 100,000 person-years) [3] and heterogeneous diseases characterized by the presence of autoantibodies that react against red blood cell self-antigens and could lead to a variety of life-threatening complications including thrombosis. After the first evidence in the 1960’s of the occurrence of pulmonary embolism in 11% of patients with AIHA [4], a hypercoagulable state has been demonstrated in these patients [5] and more and more reports have been published making thrombotic manifestations a hallmark of AIHA, as 10–20% of patients experience a thrombotic event, either arterial or venous (Table 1) [1,6,7,8,9,10,11,12,13,14,15,16]. Moreover, one fatal pulmonary embolism has been reported among 21 AIHA patients who received antithrombotic prophylaxis with low-molecular-weight heparin [6]. A review of the literature on thrombotic complications in patients with AIHA showed 60 thrombotic episodes (44 venous and 16 arterial, including deep vein thrombosis, pulmonary embolism, acute myocardial infarction, stroke and transient ischemic attack) in 474 patients (12.7%) [17]. Two single-center retrospective studies showed that eight of 40 (20%) and 11 of 48 (23%) AIHA patients had a venous thromboembolic event, respectively [10,11]. In a recent retrospective analysis of 378 AIHA patients, venous thrombosis occurred in 58 patients (15%), with no increased risk of mortality [12]. These data have been confirmed by a recent review of the literature showing a prevalence of VTE in AIHA patients between 0% and 27% [18]. Cold agglutinin disease (CAD) is a particular form of AIHA in which the pathogenic autoantibodies are represented by cold agglutinins that trigger hemolysis via classical complement pathway activation. Epidemiological data on thrombotic risk and its impact on mortality rate in patients with CAD are limited, but recently a population-based [13] and a retrospective cohort study over a 10-year period [15] reported an approximately two-fold increased risk of thrombotic events compared to non-CAD patients and a shorter follow-up, suggesting a potential increased risk of mortality in patients with thrombotic events. In a phase II study, treatment with eculizumab was associated with the absence of thromboembolic events, although the follow-up period was too short (less than one year) to draw significant conclusions [19]. In PNH, the uncontrolled activation of the complement pathway causes a chronic intravascular hemolysis [20] leading to life-threatening complications, particularly thrombosis that represents the most common cause of mortality, accounting for up to 67% of death [5,21]. A high incidence of thrombosis has been reported regardless the hemolytic status [22] and approximately 5–10% of PNH patients will present with thrombosis [7,16]. Venous thrombosis is more frequent than arterial and occurs mostly at unusual sites such as splanchnic or cerebral veins [21,23]. The last report from the International PNH Registry (NCT01374360), the largest ongoing multicenter study on PNH, showed that 544 of 4134 patients (13.2%) had a history of thrombotic events [8]. The risk of VTE in patients with PNH remains high also in patients receiving antithrombotic prophylaxis [24]. In contrast, the introduction of eculizumab as the treatment of choice for PNH determined an 85% relative risk reduction of thrombotic events in a cohort of 195 PNH patients (1.07 events/100 patient-years in treated compared to 7.37 events/100 patient-years in non-treated) [25]. This rate increased to 94% among the 56% of patients that were also anticoagulated (0.62 compared to 10.61 events/100 patient-years). The mortality rate attributable to thrombosis was reduced accordingly [26,27], although patients with thrombosis had a reduced overall survival (HR 4.48) despite treatment with eculizumab [28].

Risk factors for thrombosis seem to be particularly related to hemolysis itself, as the majority of thrombotic events have been reported during the active phases. Indeed, severe anemia (Hb < 8.5 g/dL) and high lactate dehydrogenase levels, well-known markers of hemolysis, have been associated with an increased risk of thrombosis [6,10]. In these patients, the presence of an undiagnosed malignancy (which may be associated with AIHA) or other common acquired risk factors for thrombosis, need to be considered. Antiphospholipid antibodies (APA) as well have been proposed as possible risk factors for thrombosis, but with inconclusive results [1,29]. Splenectomy is another strong risk factor for venous thrombosis, both immediately after and at 90 days after surgery, with a hazard ratio of 2.66 (95%CI 1.36–5.23) and of 3.29 (95%CI 2.10–5.16), respectively, as reported by the largest population-based study conducted so far [14].

## 3. Pathophysiology of Thrombosis in Immune Mediated Hemolysis

The association between immune-mediated hemolytic anemias and thrombosis is multifactorial, and it is driven by mechanisms that extend far beyond the activation of the immune system. It is well-established that hemolysis itself, regardless its etiology, may lead to endothelial dysfunction and activation of platelets that trigger the coagulation cascade. On the other hand, peculiar mechanisms of immune etiology are pivotal in the activation of thrombosis occurring in immune-mediated hemolytic anemias such as PNH.

### 3.1. Non-Immune Related Mechanisms of Thrombosis in Hemolytic Anemia

**Figure 1:**
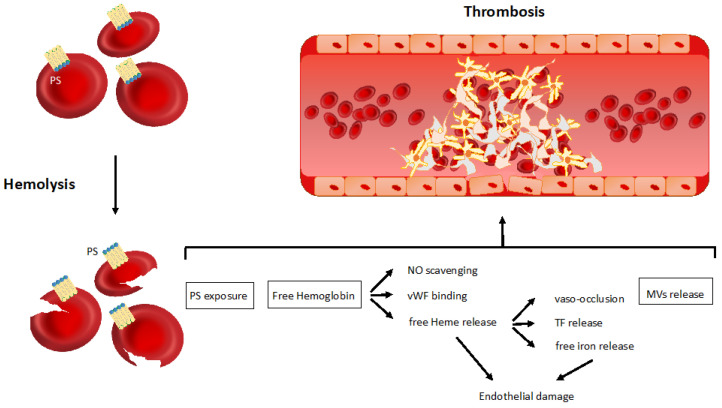
Non-immune related mechanisms of thrombosis in hemolytic anemia. The main pathophysiological mechanisms linking hemolysis to thrombosis are represented in the figure—phosphatidylserine exposure on the red blood cell surface; free hemoglobin and heme release from damaged red blood cells; red blood cells-derived microvesicle shedding. PS, phosphatidylserine; NO, nitric oxide; vWF, von Willebrand factor; TF, tissue factor; MVs, microvesicles.

Although Virchow’s triad (vessel wall abnormalities, blood flow stasis and hypercoagulability) refers to venous thrombosis, current knowledge supports its translation to arterial thrombosis as well [30]. In hemolytic anemia, all the components of Virchow’s triad are triggered, owing predominantly to non-immune mechanisms. Among them, the exposure of phosphatidylserine on the red blood cell (RBC) surface, the release of free hemoglobin and heme from damaged RBCs, and the shedding of RBC-derived microvesicles are worth mentioning (Figure 1).

#### 3.1.1. Phosphatidylserine Exposure

The activation of the coagulation cascade requires an adequate prothrombotic surface for a correct assembly of the prothrombinase complex. It is traditionally accepted that this surface is mainly provided by negatively charged phosphatidylserine on activated platelet membranes [31]. However, recent insights suggest a role also for RBCs in supplying phosphatidylserine necessary to trigger coagulation. Indeed, RBCs damaged by hemolysis may lose their membrane asymmetry, exposing phosphatidylserine, which is normally located in the inner layer of the plasma membrane [32], leading to activation of apoptotic signals for damaged cell removal. Although this mechanism has been described more deeply in the group of congenital hemolytic anemias such as beta-thalassemia and sickle cell anemia [33], a possible role has been shown also in patients with AIHA [34].

#### 3.1.2. Free Hemoglobin and Heme Release

Hemoglobin and heme are released in hemolytic anemia as products of intravascular hemolysis and lead to the activation of endothelial cells in different ways. In particular, extracellular hemoglobin acts as a strong nitric oxide (NO) scavenger, sequestering it from the circulation [35]. Normally, NO is released from the intact endothelial surface and acts as a physiological endogenous platelet inhibitor. Plasma NO depletion in hemolytic anemia may also be explained by other mechanisms unrelated to free hemoglobin. In particular, arginase, one of the enzymes released by damaged RBCs, cleaves L-arginine, reducing its bioavailability as a substrate necessary for NO synthesis [36]. Hemoglobin also reduces NO bioavailability through an indirect mechanism, preventing NO-mediated inhibition of platelet activation through the cGMP-dependent signaling and therefore resulting in enhanced platelet activation [36,37,38]. Moreover, NO plasma level reduction determines the loss of some antithrombotic functions, such as vasodilatation, the subsequent maintenance of vascular homeostasis [39] and the inhibition of cytokine-induced tissue factor expression [40].

Free hemoglobin may also increase the risk of thrombosis through the interaction with von Willebrand factor (vWF). Firstly, hemoglobin was shown to bind vWF directly, increasing the affinity of the vWF A1 domain for the glycoprotein Ib receptor on the platelet surface, thus eliciting platelet aggregation [41]. Secondly, hemoglobin binds to the vWF A2 domain, making vWF less susceptible to ADAMTS13 proteolysis and resulting in the formation of ultralarge vWF multimers with high prothrombotic potential [42].

Acute hemolysis, through free hemoglobin, may cause microvascular stasis in the visceral circulation that increases the risk of coagulation activation [43]. However, according to an in vivo study in mice, hemoglobin is indirectly responsible for the activation of this process through the release of its oxygen-binding domain heme. The latter may induce, via toll-like receptor 4, the release of prothrombotic P-selectin and vWF stored in the Weibel–Palade bodies of the endothelial cells [44]. These data were corroborated by the demonstration of the inhibition of vaso-occlusion and endothelial damage after heme inactivation by its binding to the high-affinity heme-binding protein hemopexin [44]. Furthermore, free heme may induce thrombin generation through the expression of tissue factor on the endothelial cell surface, via activation of the nuclear factor-kappa B transcription factor [45]. Heme has also been recognized as possible mediator of low-density lipoprotein oxidation, promoting endothelial cell injury and atherogenesis [46], so increasing the risk of arterial thrombosis. The role of heme in arterial thrombosis is also confirmed by preclinical studies in which hemin, a heme-oxigenase 1 inducer, through the activation of heme degradation, was protective against microvascular and carotid occlusion [47,48]. Hemoglobin, heme and especially free iron derived from damaged RBCs may also lead to endothelial impairment through the induction of the production of reactive oxygen species (ROS) that cause an abnormal expression of adhesion molecules (VCAM-1 and ICAM-1) that determine vaso-occlusion through the adhesion of RBCs, leukocytes and platelets to the endothelium. The vascular damage induced by ROS is worsened by NO reaction with superoxide, that forms peroxynitrite, a highly harmful molecule for the endothelium [49]. The resulting inflammation and hypoxia further increase tissue factor expression and the consequent activation of the coagulation cascade [50]. One of the principal consequences of this inflammatory activation caused by free heme is the release of neutrophil extracellular traps (NETs), mesh-works of chromatin fibers released by neutrophils, which favor coagulation by serving as a scaffold for the activation of platelets and coagulation factors [51]. NETs have been involved in the pathogenesis of thrombosis in other hemolytic anemias, such as sickle cell disease [52].

#### 3.1.3. Microvesicles Shedding

An increased number of circulating RBC-derived microvesicles has been reported in hemolytic anemia [53,54,55]. Microvesicles are known to be involved in a wide spectrum of biological processes including thrombosis and inflammation [56]. The release of RBC-derived microvesicles, rich in hemoglobin, may account, owing to NO scavenging, for the enhancement of a prothrombotic state in hemolytic anemia [57]. In addition, phosphatidylserine-expressing microvesicles may trigger thrombin generation through the activation of factor IX or the binding of factor X and the prothrombinase complex [54], while the direct release of tissue factor by microvesicles is debated [58]. A study on patients with sickle cells disease showed that circulating levels of RBC-derived microvesicles were strongly correlated with plasma levels of such biomarkers of thrombin generation as D-dimer and prothrombin fragment 1 + 2 [54], supporting the association between RBC-derived microvesicles and thrombosis.

### 3.2. Immune-Mediated Mechanisms of Thrombosis in Hemolytic Anemia

Autoimmune diseases are associated with an increased risk of venous and arterial thrombosis [59] as they are characterized by an enhanced inflammatory activity, which accounts for a prothrombotic state through several mechanisms, such as cytokine-induced tissue factor expression, endothelial dysfunction, inhibition of the protein C system and fibrinolysis (through the increase of the plasma levels of plasminogen activator inhibitor 1), platelet activation, release of microvesicles, NET generation, activation of the contact system and presence of increased levels of fibrinogen, vWF and factor VIII [51,59,60,61,62,63].

#### 3.2.1. Autoimmune Hemolytic Anemia

The existence of peculiar mechanisms underlying the development of thrombosis in AIHA, in addition to those already mentioned, is not completely clear. However, it is widely accepted that the main mechanism underlying thrombosis in AIHA is the autoantibody-induced RBC membrane alteration that leads to hemolysis [64,65]. In addition, in line with the common association between autoimmune diseases and APA [66], up to 60% of patients with AIHA develop APA, whose positivity is a well-known risk factor for both arterial and venous thrombosis [1,67,68]. Hence, one would expect that, during acute hemolysis, the presence of APA further unbalances the hemostatic equilibrium. However, data on this topic are controversial and several studies failed to prove an association between APA positivity and thrombosis in AIHA [10,11,69]. The observation that the vast majority of thromboembolic events occur during flares of active hemolysis [1,6,9,10] highlights that hemolysis itself, rather than disease-specific immune-mediated mechanisms, possibly represents the most important pathophysiological mechanism of the prothrombotic state in AIHA.

On the other hand, following the observation that in most AHIA patients active hemolysis is associated with complement activation [9], similarly to what has been suggested for PNH, it is possible that complement may play a role in thrombotic event development in patients with AIHA as well. Indeed, an intensive crosstalk between the complement system and the coagulation cascade exists [70]. The complement system is activated via different pathways (i.e., classical, alternate and lectin pathway) all resulting in the formation of convertases leading to the development of the active forms of the complement components, C3a/C3b and C5a/C5b. C5a and C5b initiate the complement terminal pathway [71], resulting in the formation of the membrane attack complex (MAC). Several mechanisms may account for complement-mediated coagulation activation. In particular, C5a and C5b activate platelets [72], induce tissue factor expression [73], stimulate vWF [74] and P-selectin release [75], and induce exposure of prothrombinase assembly sites on endothelial cells [74]. Furthermore, the key enzyme of the lectin pathway, MASP-2, is able to directly activate thrombin generation via prothrombin cleavage [76]. Moreover, C5a induces the transition from profibrinolytic to prothrombotic activities on basophils and mast cells through the upregulation of plasminogen activator inhibitor-1 (PAI-1) [77]. While in warm autoimmune hemolytic anemia the complement system is hardly essential for hemolysis, the latter may be considered predominantly complement-mediated in cold agglutinin disease [78]. IgM cold agglutinins, bound to erythrocytes cell surface antigens, directly interact with C1q, triggering the activation of the classical complement pathway, bringing C5 activation before its cleavage into C5a and C5b, formation of the MAC and eventually intravascular hemolysis [79].

#### 3.2.2. Paroxysmal Nocturnal Hemoglobinuria

The association between PNH and thrombosis is still not completely understood, allegedly involving a combination of hemolytic and non-hemolytic mechanisms. Among the latter, the interaction between platelets, the complement system and coagulation is likely to explain the thrombotic burden of PNH and its related mortality. By initiating clot formation, platelets have been reported to play a pivotal role in thrombosis among patients with PNH [80]. Despite the fact that deficiency of the cell surface regulatory proteins CD55 and CD59 makes platelets susceptible to complement attack, potentially resulting in their removal from circulation and consequent thrombocytopenia [81], the lifespan of PNH platelets is normal [82]. Instead of platelet destruction, complement activation observed in PNH starts a process that triggers platelet shape change, through the formation of the membrane attack complex (C5b–9) on the platelet surface, culminating in platelet activation [83]. Activated platelets secrete the content of α-granules that, through membrane depolarization, may fuse with the platelet membrane [84], enabling the exocytosis of a vesiculated membrane attack complex and the release of prothrombotic platelet-derived microvesicles [85]. Exposure of phosphatidylserine on the microvesicle surface acts as a binding site for prothrombinase complex and factor X, triggering thrombin generation [86]. Activated platelets may also interact with neutrophils, promoting thrombosis through the release of neutrophil serine proteases and nucleosomes (NETosis), with the activation of factor X [87]. Several studies addressed the role of fibrinolysis in the development of thrombosis in PNH. The impairment of fibrinolysis seems to be related to the deficiency of the GPI-anchored urokinase plasminogen activator (u-PA) receptor (u-PAR or CD87) in PNH leukocytes and platelets [88]. In the absence of the GPI anchor, u-PAR cannot bind to the cell membrane, being released in plasma where it competes with membrane-bound u-PAR for the binding of u-PA [89]. This process leads to a decreased availability of u-PA, resulting in a reduced conversion of plasminogen into plasmin and thus a reduced fibrinolysis. A recent study showed that also patients with PNH and asymptomatic hemolysis had an increased risk of thrombosis [90]. Patients with the higher risk of thrombosis were those with the highest levels of PNH white cells, implying for white blood cells a role in the development of thrombosis in PNH, possibly because of their role in NET generation.

## 4. Thrombophilia in Immune-Mediated Hemolysis

The term thrombophilia refers to the inherited or acquired coagulation abnormalities associated with an increased risk of venous thrombosis. Inherited thrombophilia abnormalities recognized as independent risk factors for venous thromboembolism are represented by the common gain-of-function mutations in the coagulation factors V (G1691A, factor V Leiden, FVL) and II (prothrombin G20210A) and the rare lack-of-function deficiencies in the natural anticoagulant proteins antithrombin (AT), protein C (PC), and protein S (PS) [91]. The most common acquired thrombophilia abnormality is represented by the presence of APA, defined by positivity of at least one test among clot-based assays for lupus anticoagulant (LAC) (partial thromboplastin time, dilute Russell’s viper venom time, silica clotting time) and enzyme-linked immunosorbent assay (ELISA)-based tests for IgG and IgM anticardiolipin (ACA) and anti-beta2glycoprotein1 antibodies [92].

No data are available on the prevalence of inherited thrombophilia abnormalities in AIHA, while the role of APA has been investigated in several studies, with controversial results. The first report showed an approximately four-fold increased risk of VTE in a series of 41 patients with AIHA associated with systemic lupus erythematosus [29]. This association was further supported by a prospective study on 30 AIHA patients, showing a 63% prevalence of APA (LAC and ACA) and a 7.5-fold increase risk of VTE for patients with LAC, while no significant association was found with ACA positivity [1]. However, other studies did not confirm the association. In particular, six of 28 AIHA patients who developed thrombosis had no detectable APA [6]. The largest retrospective study reported a 13% prevalence of APA (ACA or LAC) in 308 AIHA patients, but none of the 33 with VTE were positive [9]. In a single-center retrospective study of 40 AIHA patients, APA was detected in four patients but in none of the eight with VTE [10]. Two further similar studies failed to find LAC or ACA or anti-beta2glicoprotein1 in 21 AIHA patients tested out of 23 who developed VTE [11,69].

In view of the aforementioned factors, considering the high risk of VTE associated with PNH, the coexistence of thrombophilia abnormalities may further increase such risk. The first study investigated FVL in 56 PNH patients, showing similar prevalence as in healthy volunteers (1.8% vs. 2.8%, respectively) [93]. Another study described 16 PNH patients, with two of the four patients who developed thrombosis having an inherited thrombophilia (heterozygous FVL), compared to only one (heterozygous prothrombin gene mutation) of the remaining 12 patients without VTE [94]. The presence of inherited (AT, PC, PS, FVL, prothrombin G20210A mutation) and acquired (LAC and ACA) thrombophilia was evaluated in 13 PNH patients and in 100 healthy controls, showing similar prevalence except for APA, which had more cases in patients than controls [95]. The last published study showed no difference in plasma levels of PC and PS in PNH patients, with and without VTE [96].

Overall, the clinical utility of thrombophilia testing on therapeutic decisions is still debated and no guidelines have been published so far. However, in particular clinical settings in which there is no clear indication to antithrombotic prophylaxis, thrombophilia testing may add useful information for a global evaluation of the thrombotic risk and may help in the decision-making for the use of antithrombotic prophylaxis.

## 5. Diagnostic-Therapeutic Approach for Thrombosis in Immune-Mediate Hemolysis: Expert Opinion from a Tertiary Referral Center

Increasing evidence is available on the high incidence of thrombosis in AIHA, although in clinical practice VTE is still an underappreciated complication. Clinical presentation of patients with severe anemia due to acute-onset hemolysis is similar to that of patients with pulmonary embolism, i.e., chest pain and dyspnea, due to cardiac ischemia or pulmonary hypertension. Hence, a particular attention to signs and symptoms suggestive of VTE should be paid, for a prompt diagnosis and appropriate treatment.

### 5.1. Treatment of VTE

Because VTE occurs more likely during hemolysis, its onset is usually associated with severe anemia. This may represent a contraindication to start an anticoagulant therapy at therapeutic doses. Indeed, anemia itself is a well-known risk factor for bleeding [97] and, at the same time, it has been suggested to be associated with thrombosis independently of the determining cause [98,99]. Moreover, considering that anemia is caused by hemolysis rather than blood loss, anticoagulant treatment is not expected to worsen the degree of anemia unless concomitant hemorrhagic risk factors are present. Hence, in case of onset of VTE concomitant with an acute hemolysis, anticoagulant therapy should be promptly started, regardless the degree of anemia.

**Figure 2:**
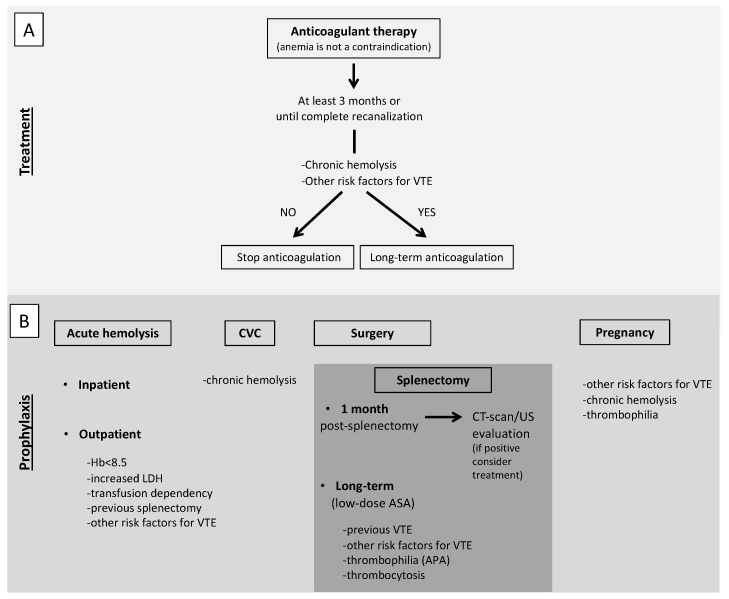
Proposed recommendations for treatment (**A**) and prophylaxis (**B**) of VTE in AIHA patients. (**A**). Anticoagulant therapy should be promptly started, regardless the degree of anemia, and continued for at least 3 months or until complete recanalization. Even if complete recanalization is reached, long-term anticoagulation should be considered for secondary prophylaxis of VTE, evaluating the presence of persistent risk factors for VTE. (**B**). Antithrombotic prophylaxis with low-molecular-weight heparin is recommended for inpatients with acute hemolysis and should be considered for outpatients with acute hemolysis associated with severe anemia, particularly in splenectomized patients or in the presence of concomitant risk factors for VTE. In patients with acute hemolysis undergoing central venous catheter positioning, antithrombotic prophylaxis should be started. We suggest antithrombotic prophylaxis for all patients with chronic immune-mediated hemolysis. In patients undergoing splenectomy, post-operative antithrombotic prophylaxis with low-molecular-weight heparin should be started, unless contraindicated and continued for at least one month when an objective evaluation should be performed before its withdrawal. Long-term anticoagulation or low-dose aspirin should be taken into account in patients with previous VTE or concomitant risk factors for VTE. In pregnant women with chronic hemolysis, antithrombotic prophylaxis should be started, especially in the presence of other risk factors of VTE or thrombophilia abnormalities. VTE, venous thromboembolism; AIHA autoimmune hemolytic anemia; CVC, central venous catheter; CT-scan, computed tomography scan; US, ultrasound; ASA, acetylsalicylic acid; APA, antiphospholipid antibodies.

In AIHA patients with VTE, anticoagulant therapy should be continued for at least 3 months or until complete recanalization [100]. Even if complete recanalization is reached, long-term anticoagulation should be considered for secondary prophylaxis of VTE. To make this decision, an extensive analysis of persistent risk factors for VTE should be done. In particular, a chronic hemolysis together with other risk factors for VTE, such as the use of steroids and thrombophilia abnormalities (particularly the presence of APA), should prompt to consider long-term anticoagulation, possibly at reduced doses (Figure 2, panel A). Considering the role of complement in specific subtypes of AIHA, namely CAD, treatment with eculizumab should be taken into account, based on the results of a phase II study that showed the absence of thromboembolic events in patients treated with eculizumab [19].

**Figure 3:**
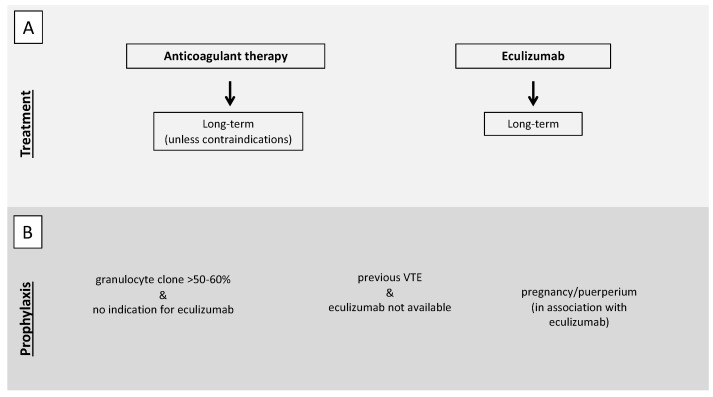
Proposed recommendations for treatment (**A**) and prophylaxis (**B**) of VTE in PNH patients. (**A**). In the case of VTE, both anticoagulant therapy and eculizumab should be immediately started. Anticoagulant therapy should be continued long-term, independently of concomitant therapy with eculizumab, unless contraindications are present such as bleeding or severe thrombocytopenia. (**B**) Antithrombotic prophylaxis should be considered in patients with a granulocyte clone >50–60% not fulfilling indication for eculizumab therapy, in patients with previous VTE in geographical areas in which eculizumab is not available, and during pregnancy and puerperium. VTE, venous thromboembolism; PNH paroxysmal nocturnal hemoglobinuria.

As for PNH patients, other than anticoagulant therapy, eculizumab should be immediately started [101] to reduce the principal driver of the prothrombotic state represented by an activated complement system. In PNH patients, the concomitant presence of thrombocytopenia secondary to bone marrow failure (one of the features of the PNH triad together with hemolysis and thromboembolism) makes anticoagulant therapy particularly challenging. PNH patients with a history of VTE should continue anticoagulant therapy long-term, independently of concomitant therapy with eculizumab [101], unless contraindications are present such as bleeding or severe thrombocytopenia. Only a few case reports are available on anticoagulation withdrawal after reaching disease control with eculizumab. However, the evidence of an 85% relative risk reduction of thrombotic events in patients on eculizumab [21] raises the option to reserve long-term anticoagulation for patients with other concomitant risk factors for thrombosis (Figure 3, panel A) [102].

### 5.2. Antithrombotic Prophylaxis

Primary antithrombotic prophylaxis is a challenging issue. VTE is more common during acute hemolysis with an incidence rate of approximately 20%, particularly if associated with severe anemia (hemoglobin less than 8.5 g/dL), high lactate dehydrogenase levels and other hemolysis parameters, transfusion-dependency and previous splenectomy [6,10,103]. According to the 2017 BJH guidelines and the 2020 International Consensus Meeting on AIHA, an antithrombotic prophylaxis with low-molecular-weight heparin is recommended for inpatients with acute hemolysis (grade 1C and 99% agreement respectively) and should be considered for outpatients with acute hemolysis associated with severe anemia (Hb < 8.5 g/dL) (grade 2C and 97% agreement, respectively) [103,104], particularly in the presence of concomitant risk factors for VTE, such as previous events, reduced mobility, thrombophilia abnormalities, age, active cancer. Another particular scenario is central venous catheter positioning that represents a local risk factor for venous thrombosis but it is not an indication for primary prophylaxis per se. Our suggestion is to start an antithrombotic prophylaxis in patients with active hemolysis undergoing central venous catheter positioning (Figure 2, panel B). A lower incidence of VTE in patients on prophylaxis during a hemolytic flare (1/21, 4.8%) compared to those without prophylaxis (5/15, 33.3%) has been reported [6]. For patient candidates for long-term primary antithrombotic prophylaxis, low-dose aspirin has been proposed other than low-molecular-weight heparin [2,105].

In PNH patients with a granulocyte clone >50–60% not fulfilling the indication for eculizumab therapy, primary antithrombotic prophylaxis should be taken into account [101], according to studies in the pre-eculizumab era showing a high risk of VTE with larger clones (35–54% vs 6–17%) [16,106,107]. Primary antithrombotic prophylaxis should be considered for PNH patients with previous VTE in geographical areas in which eculizumab is not available, whereas it is not indicated for patients without previous VTE who start treatment with eculizumab. The 85% relative risk reduction of thrombotic events in patients on eculizumab [21] prompt us to avoid antithrombotic prophylaxis in these patients in the absence of other risk factors for thrombosis (Figure 3, panel B) [102].

Arterial thrombotic events are less frequent than venous. In two large studies on AIHA patients, a total of eight (2%) arterial thrombosis (three strokes, two transient ischemic attack, three cardiac ischemic events) occurred [9,12]. Secondary prophylaxis with antiplatelet drugs in patients with previous arterial disease appears prudent.

#### 5.2.1. Particular Situation: Splenectomy

Another condition in which antithrombotic prophylaxis should be considered is surgery. Due to the high risk of VTE associated with surgery itself and the consequent reduced mobility, we suggest antithrombotic prophylaxis for all patients with chronic immune-mediated hemolysis independently of the presence of other concomitant risk factors for VTE. A specific situation is represented by splenectomy, that is an independent risk factor for thrombosis in patients with immune-mediate hemolysis [9], mostly affecting the splenic but also the portal vein. Around 2% of patients undergoing splenectomy for different causes develop a VTE within 90 days of the procedure [108,109] with the highest risk in patients with immune-mediated hemolysis (prevalence as high as 8% in one series) [110]. After splenectomy, both systemic and local factors may contribute to the development of thrombosis. Systemic factors are represented by the hypercoagulable state induced by surgery and reactive thrombocytosis secondary to spleen removal, while the local factor is represented by ligation of the residual splenic vein with formation of a stump of the vein characterized by blood stasis and turbulence, resulting in increased local coagulability. Based on this evidence, any suggestive sign or symptom of splanchnic vein thrombosis after a splenectomy, such as abdominal pain, fever or ileus, should prompt to perform an objective diagnosis. Moreover, an appropriate post-operative antithrombotic prophylaxis with low-molecular-weight heparin should be started, unless contraindicated (grade of recommendation 1C in the 2017 BJH guidelines [104], and an extended period after hospital discharge should be considered for patients at particularly high risk (grade of recommendation 1C in the 2017 BJH guidelines [104,111]. Although the benefit of an extended antithrombotic prophylaxis is uncertain, we suggest to continue for at least one month after splenectomy and to perform objective testing before its withdrawal. Since the long-term risk of VTE is increased after splenectomy, due to such local or systemic causes as a closed splenic vein or reactive thrombocytosis, particularly in patients with immune-mediated hemolysis, long-term anticoagulant or low-dose aspirin prophylaxis should be taken into account, particularly in patients with previous VTE or concomitant risk factors such as thrombophilia abnormalities (mainly APA) (Figure 2, panel B). Although a systematic testing for thrombophilia is not recommended so far in all patients with immune-mediate hemolysis, the presence of APA should be assessed before splenectomy [69].

#### 5.2.2. Particular Situation: Pregnancy

Pregnancy, because of the physiological procoagulant changes in the hemostatic balance, is a situation at high risk of VTE per se. In the case of immune-mediated hemolysis, antithrombotic prophylaxis may be considered. Considering its extreme rarity, no evidence is available on how to manage pregnant women with immune-mediated hemolysis. However, the 2017 BJH guidelines suggest to consider antenatal and 6 weeks postnatal prophylaxis if other risk factors coexist (grade of recommendation 1C) (Figure 2, panel A) [104]. In this context, a thrombophilia screen may help in the decision to prescribe antithrombotic prophylaxis. As for PNH, the immune-mediated hemolytic anemia with the most severe prothrombotic state, antithrombotic prophylaxis with LMWH should be prescribed in pregnancy and puerperium in the absence of contraindications. The 2019 Consensus Statement of the Canadian PNH Network suggests the use of eculizumab in pregnancy with a close follow-up, especially after the first trimester, as over 50% of patients require an increased dose and/or frequency of eculizumab until delivery. In the pre-eculizumab era, maternal mortality was around 8–21%, mostly due to VTE, with a fetal mortality of 4–9%, while no maternal deaths and a fetal mortality of 4% were reported in a real-life study on the use of eculizumab in pregnancy. In this study, 88% of patients were also anticoagulated and no VTE occurred during pregnancy but two (3%, one deep vein thrombosis and one splanchnic venous thrombosis) during the post-partum period and other two after eculizumab discontinuation (Figure 3, panel B) [101,112]. A similar consideration can be made for women who use oral contraceptives that are associated with an increased risk of thrombosis. Although a thrombophilia screen in the general population before their prescription is not advised, in these patients it can optimize their use and lead to the prescription of compounds associated with a low risk of thrombosis or to prescribe concomitant anticoagulant therapy in selected cases.

## 6. Conclusions

VTE is an important cause of morbidity and mortality in patients affected by an immune-mediated hemolytic anemia and requires a prompt diagnosis and an appropriate treatment. Although management strategies have been better defined in the last years for patients with an acute hemolysis, evidence is still lacking on the optimal use of antithrombotic prophylaxis in other situations such as chronic hemolysis, invasive procedures, splenectomy and pregnancy. In these situations, a careful and thorough evaluation of the individual risk profile of VTE will help to identify patients at increased risk who may benefit from antithrombotic prophylaxis. To date, few data are available on thrombophilia abnormalities in patients with immune-mediated hemolysis and those on APA are inconsistent. However, because of their high prothrombotic risk, it is advised to test all patients at least for APA. A wide thrombophilia screen can be considered in the above-mentioned specific situations to optimize the decision on primary antithrombotic prophylaxis.

## Figures and Tables

**Table 1:** Most relevant studies on thrombotic risk in AIHA, CAD and PNH. Where possible, the site of thrombosis and the use of antithrombotic prophylaxis have been reported as well. AIHA, autoimmune hemolytic anemia; CAD, cold agglutinin disease; PNH, paroxysmal nocturnal hemoglobinuria; DVT, deep vein thrombosis; PE, pulmonary embolism; NA, not available; MI, myocardial infarction; CVT, cerebral vein thrombosis; TIA, transient ischemic attack; DIC, disseminated intravascular coagulation; LMWH, low molecular weight heparin; VKA, vitamin K antagonists; VTE, venous thromboembolism.

Study	Design	Patients	Thrombotic Events	Prothrombotic Risk Factors	Antithrombotic Prohpylaxis
Pullarkat et al., 2002 [1]	Multicenter Prospective	30 AIHA from 1995 to 2001	8 (27%) with at least one event (7 DVT, 3 PE, 1 PE + portal vein thrombosis)	Lupus anticoagulant Active hemolysis (4/8)	NA
Hendrick et al., 2003 [6]	Single-center Retrospective	28 (23 AIHA + 5 CAD) from 1986 to 2001	6 (21%) (4 PE, 1 MI, 1 CVT)	NA	21 (1 of whom developed a fatal PE)
Barcellini et al., 2014 [9]	Multicenter Retrospective	308 (224 AIHA + 84 CAD) from 1978 to 2013	33 (11%) with at least one event (13 DVT, 11 PE, 5 splanchnic, 3 strokes, 2 TIA, 3 cardiac ischemic events, 1 DIC)	Hemoglobin < 6 g/dL Intravascular hemolysis Previous splenectomy	NA
Lecouffe-Desprets et al., 2015 [10]	Single-center Retrospective	40 AIHA from 2009 to 2013	8 (20%) (4 PE, 4 DVT + PE)	Low hemoglobin levels Active hemolysis (7/8)	0/8
Audia et al., 2018 [11]	Single-center Retrospective	48 AIHA from 2006 to 2016	11 (23%) (5 DVT, 1 PE, 5 DVT + PE)	Active hemolysis (10/11)	2/11 (LMWH prophylaxis, VKA)
Barcellini et al., 2018 [12]	Multicenter Retrospective	378 (271 AIHA + 107 CAD)	58 (15%) (14 with CAD)	NA	NA
Bylsma et al., 2019 [13]	Multicenter Retrospective (population-based registry)	72 CAD from 1999 to 2013	7 (10%) with at least one event (venous or arterial)	NA	NA
Ho G et al., 2020 [14]	Multicenter Retrospective (population-based registry)	4756 AIHA from 1991 to 2014	259 (5%) VTE (61 splanchnic)	Splenectomy	NA
Broome et al., 2020 [15]	Multicenter Retrospective (population-based registry)	608 CAD from 2006 to 2016	180 (30%) (19 DVT, 30 PE, 12 splanchnic, 57 other VTE, 85 cerebral events, 13 arterial thrombosis, 34 MI)	NA	NA
Hall et al., 2003 [16]	Single-center Prospective	163 PNH	29 (18%) with at least one event (20 splanchnic, 4 DVT, 3 PE, 3 CVT, 1 retinal, 1 catheter related thrombosis)	NA	32 patients on primary prophylaxis with warfarin (no VTE)
De Latour et al., 2008 [7]	Multicenter Retrospective	454 PNH from 1950 to 2005	116 (26%) with at least one event (31 DVT, 35 central nervous system, 49 Budd-Chiari syndrome, 29 others)	Age >55 years Use of transfusion Previous thrombosis Warfarin as primary prophylaxis	18 patients on primary prophylaxis with warfarin (10 VTE) 19 patients on secondary prophylaxis with warfarin (8 VTE)
Schrezenmeier et al., 2020 [8]	Multicenter prospective registry	4134 PNH from 2007 to 2017	779 (19%) (SVT, DVT, splanchnic vein or arterial thrombosis, acute peripheral vascular disease occlusion, CVT, PE, MI, TIA, stroke)	Hemolysis, regardless of clone size	NA

## Data Availability

No new data were created or analyzed in this study. Data sharing is not applicable to this article.

## References

[1] Pullarkat V., Ngo M., Iqbal S., Espina B., Liebman H.A. (2002). Detection of lupus anticoagulant identifies patients with autoimmune hae-molytic anaemia at increased risk for venous thromboembolism. Br. J. Haematol..

[2] L’Acqua C., Hod E. (2015). New perspectives on the thrombotic complications of haemolysis. Br. J. Haematol..

[3] Hansen D.L., Möller S., Andersen K., Gaist D., Frederiksen H. (2020). Increasing incidence and prevalence of acquired hemolytic anemias in Denmark, 1980–2016. Clin. Epidemiol..

[4] Allgood J.W., Chaplin H. (1967). Idiopathic acquired autoimmune hemolytic anemia. A review of forty-seven cases treated from 1955 through 1965. Am. J. Med..

[5] Chapin J., Terry H.S., Kleinert D., Laurence J. (2016). The role of complement activation in thrombosis and hemolytic anemias. Transfus. Apher. Sci..

[6] Hendrick A. (2003). Auto-immune haemolytic anaemia—A high-risk disorder for thromboembolism?. Hematology.

[7] de Latour R.P., Mary J.Y., Salanoubat C., Terriou L., Etienne G., Mohty M., Roth S., de Guibert S., Maury S., Cahn J.Y. (2008). Paroxysmal nocturnal hemoglobinuria: Natural history of disease subcategories. Blood.

[8] Schrezenmeier H., Röth A., Araten D.J., Kanakura Y., Larratt L., Shammo J.M., Wilson A., Shayan G., Maciejewski J.P. (2020). Baseline clinical characteristics and disease burden in patients with paroxysmal nocturnal hemoglobinuria (PNH): Updated analysis from the International PNH Registry. Ann. Hematol..

[9] Barcellini W., Fattizzo B., Zaninoni A., Radice T., Nichele I., Di Bona E., Lunghi M., Tassinari C., Alfinito F., Ferrari A. (2014). Clinical heterogeneity and predictors of outcome in primary autoimmune hemolytic anemia: A GIMEMA study of 308 patients. Blood.

[10] Lecouffe-Desprets M., Neel A., Graveleau J., Leux C., Perrin F., Visomblain B., Artifoni M., Masseau A., Connault J., Pottier P. (2015). Venous thromboembolism related to warm auto-immune hemolytic anemia: A case-control study. Autoimmun. Rev..

[11] Audia S., Bach B., Samson M., Lakomy D., Bour J.-B., Burlet B., Guy J., Duvillard L., Branger M., Leguy-Seguin V. (2018). Venous thromboembolic events during warm autoimmune hemolytic anemia. PLoS ONE.

[12] Barcellini W., Zaninoni A., Fattizzo B., Giannotta J.A., Lunghi M., Ferrari A., Leporace A.P., Maschio N., Scaramucci L., Cantoni S. (2018). Predictors of refractoriness to therapy and healthcare resource utilization in 378 patients with primary autoimmune hemolytic anemia from eight Italian reference centers. Am. J. Hematol..

[13] Bylsma L.C., Ording A.G., Rosenthal A., Öztürk B., Fryzek J.P., Arias J.M., Röth A., Berentsen S. (2019). Occurrence, thromboembolic risk, and mortality in Danish patients with cold agglutinin disease. Blood Adv..

[14] Ho G., Brunson A., Keegan T.H., Wun T. (2020). Splenectomy and the incidence of venous thromboembolism and sepsis in patients with autoimmune hemolytic anemia. Blood Cells Mol. Dis..

[15] Broome C.M., Cunningham J.M., Mullins M., Jiang X., Bylsma L.C., Fryzek J.P., Rosenthal A. (2020). Increased risk of thrombotic events in cold agglutinin disease: A 10-year retrospective analysis. Res. Pract. Thromb. Haemost..

[16] Hall C., Richards S., Hillmen P. (2003). Primary prophylaxis with warfarin prevents thrombosis in paroxysmal nocturnal hemoglobinuria (PNH). Blood.

[17] Ruggeri M., Rodeghiero F. (2015). Thrombotic risk in patients with immune haemolytic anaemia. Br. J. Haematol..

[18] Solari D., Alberio L., Ribi C., Grandoni F., Stalder G. (2021). Autoimmune hemolytic anemia and pulmonary embolism: An association to consider. TH Open.

[19] Röth A., Bommer M., Hüttmann A., Herich-Terhürne D., Kuklik N., Rekowski J., Lenz V., Schrezenmeier H., Dührsen U. (2018). Eculizumab in cold agglutinin disease (DECADE): An open-label, prospective, bicentric, nonrandomized phase 2 trial. Blood Adv..

[20] Parker C., Omine M., Richards S., Nishimura J.I., Bessler M., Ware R., Hillmen P., Luzzatto L., Young N., Kinoshita T. (2005). Diagnosis and management of paroxysmal nocturnal hemoglobinuria. Blood.

[21] Hill A., Kelly R.J., Hillmen P. (2013). Thrombosis in paroxysmal nocturnal hemoglobinuria. Blood.

[22] Yenerel M.N., Muus P., Wilson A., Szer J. (2017). Clinical course and disease burden in patients with paroxysmal nocturnal hemoglobinuria by hemolytic status. Blood Cells Mol. Dis..

[23] Ziakas P.D., Poulou L.S., Rokas G.I., Bartzoudis D., Voulgarelis M. (2007). Thrombosis in paroxysmal nocturnal hemoglobinuria: Sites, risks, outcome. An overview. J. Thromb. Haemost..

[24] Hill A., Reid S.A., Rother R.P., Gladwin M.T., Collinson P.O., Gaze D.C., Lowe A., Guthrie A., Sivananthan M.U., Hillmen P. (2006). High definition contrast-enhanced MR imaging in paroxysmal nocturnal hemoglobinuria (PNH) suggests a high frequency of subclinical thrombosis. Blood.

[25] Hillmen P., Muus P., Dührsen U., Risitano A.M., Schubert J., Luzzatto L., Schrezenmeier H., Szer J., Brodsky R.A., Hill A. (2007). Effect of the complement inhibitor eculizumab on thromboembolism in patients with paroxysmal nocturnal hemoglobinuria. Blood.

[26] Socié G., Caby-Tosi M., Marantz J.L., Cole A., Bedrosian C.L., Gasteyger C., Mujeebuddin A., Hillmen P., Walle J.V., Haller H. (2019). Eculizumab in paroxysmal nocturnal haemoglobinuria and atypical haemolytic uraemic syndrome: 10-year pharmacovigilance analysis. Br. J. Haematol..

[27] Brodsky R.A. (2021). How I treat paroxysmal nocturnal hemoglobinuria. Blood.

[28] Loschi M., Porcher R., Barraco F., Terriou L., Mohty M., De Guibert S., Mahe B., Lemal R., Dumas P.-Y., Etienne G. (2016). Impact of eculizumab treatment on paroxysmal nocturnal hemoglobinuria: A treatment versus no-treatment study. Am. J. Hematol..

[29] Kokori S.I., Ioannidis J.P., Voulgarelis M., Tzioufas A.G., Moutsopoulos H.M. (2000). Autoimmune hemolytic anemia in patients with systemic lupus erythematosus. Am. J. Med..

[30] Sanchez C.J. (2004). Venous and arterial thrombosis: A continuous spectrum of the same disease?. Eur. Heart J..

[31] Lentz B.R. (2003). Exposure of platelet membrane phosphatidylserine regulates blood coagulation. Prog. Lipid Res..

[32] Wesseling M.C., Wagner-Britz L., Huppert H., Hanf B., Hertz L., Nguyen D.B., Bernhardt I. (2016). Phosphatidylserine exposure in human red blood cells depending on cell age. Cell. Physiol. Biochem..

[33] Ataga K.I., Cappellini M.D., Rachmilewitz E.A. (2007). β-Thalassaemia and sickle cell anaemia as paradigms of hypercoagulability. Br. J. Haematol..

[34] Bartolmäs T., Mayer B., Balola A.H., Salama A. (2017). Eryptosis in autoimmune haemolytic anaemia. Eur. J. Haematol..

[35] Jeffers A., Gladwin M.T., Kim-Shapiro D.B. (2006). Computation of plasma hemoglobin nitric oxide scavenging in hemolytic anemias. Free Radic. Biol. Med..

[36] Helms C.C., Marvel M., Zhao W., Stahle M., Vest R., Kato G.J., Lee J.S., Christ G., Gladwin M.T., Hantgan R.R. (2013). Mechanisms of hemolysis-associated platelet activation. J. Thromb. Haemost..

[37] Radomski M., Palmer R.M., Moncada S. (1987). Endogenous nitric oxide inhibits human platelet adhesion to vascular endothelium. Lancet.

[38] Gambaryan S., Subramanian H., Kehrer L., Mindukshev I., Sudnitsyna J., Reiss C., Rukoyatkina N., Friebe A., Sharina I., Martin E. (2016). Erythrocytes do not activate purified and platelet soluble guanylate cyclases even in conditions favourable for NO synthesis. Cell Commun. Signal..

[39] Yetik-Anacak G., Catravas J.D. (2006). Nitric oxide and the endothelium: History and impact on cardiovascular disease. Vasc. Pharmacol..

[40] Yang Y., Loscalzo J. (2000). Regulation of tissue factor expression in human microvascular endothelial cells by nitric oxide. Circulation.

[41] Da Q., Teruya M., Guchhait P., Teruya J., Olson J.S., Cruz M.A. (2015). Free hemoglobin increases von Willebrand factor-mediated platelet adhesion in vitro: Implications for circulatory devices. Blood.

[42] Zhou Z., Hyojeong H., Cruz M.A., Lopez J.A., Dong J.-F., Guchhait P. (2008). Hemoglobin blocks von willebrand factor proteolysis by ADAMTS-13: A mechanism associated with acquired ADAMTS-13 deficiency in patients with sickle cell disease. Blood.

[43] Effenberger-Neidnicht K., Bornmann S., Jägers J., Patyk V., Kirsch M. (2019). Microvascular stasis and hemolysis: Coincidence or causality?. J. Inflamm. Res..

[44] Belcher J.D., Chen C., Nguyen J., Milbauer L., Abdulla F., Alayash A.I., Smith A., Nath K.A., Hebbel R.P., Vercellotti G.M. (2014). Heme triggers TLR4 signaling leading to endothelial cell activation and vaso-occlusion in murine sickle cell disease. Blood.

[45] Setty B.N.Y., Betal S.G., Zhang J., Stuart M.J. (2008). Heme induces endothelial tissue factor expression: Potential role in hemostatic activation in patients with hemolytic anemia. J. Thromb. Haemost..

[46] Balla G., Jacob H.S., Eaton J.W., Belcher J.D., Vercellotti G.M. (1991). Hemin: A possible physiological mediator of low density lipoprotein oxidation and endothelial injury. Arterioscler. Thromb. Vasc. Biol..

[47] Lindenblatt N., Bordel R., Schareck W., Menger M., Vollmar B. (2004). Vascular heme oxygenase-1 induction suppresses microvascular thrombus formation in vivo. Arter. Thromb. Vasc. Biol..

[48] Peng L., Mundada L., Stomel J.M., Liu J.J., Sun J., Yet S.-F., Fay W.P. (2004). Induction of heme oxygenase-1 expression inhibits platelet-dependent thrombosis. Antioxid. Redox Signal..

[49] Ronson R.S., Nakamura M., Vinten-Johansen J. (1999). The cardiovascular effects and implications of peroxynitrite. Cardiovasc. Res..

[50] Lizarralde-Iragorri M.A., Shet A.S. (2020). Sickle cell disease: A paradigm for venous thrombosis pathophysiology. Int. J. Mol. Sci..

[51] Kimball A.S., Obi A.T., Diaz J.A., Henke P.K. (2016). The emerging role of NETs in venousthrombosis and immunothrombosis. Front. Immunol..

[52] Gladwin M.T., Ofori-Acquah S.F. (2014). Erythroid DAMPs drive inflammation in SCD. Blood.

[53] Westerman M., Pizzey A., Hirschman J., Cerino M., Weil-Weiner Y., Ramotar P., Eze A., Lawrie A., Purdy G., Mackie I. (2008). Microvesicles in haemoglobinopathies offer in-sights into mechanisms of hypercoagulability, haemolysis and the effects of therapy. Br. J. Haematol..

[54] van Beers E.J., Schaap M.C., Berckmans R.J., Nieuwland R., Sturk A., van Doormaal F.F., Meijers J.C., Biemond B.J. (2009). Circulating erythrocyte-derived microparticles are associated with coagulation activation in sickle cell disease. Haematologica.

[55] Barcellini W., Zaninoni A., Giannotta J.A., Merati G., Capecchi M., Fattizzo B., Trombetta E., Artoni A. (2021). Circulating extracellular vesicles and cytokines in congenital and acquired hemolytic anemias. Am. J. Hematol..

[56] Diamant M., Tushuizen M.E., Sturk A., Nieuwland R. (2004). Cellular microparticles: New players in the field of vascular disease?. Eur. J. Clin. Investig..

[57] Reiter C.D., Wang X., Tanus-Santos J.E., Hogg N., Cannon R.O., Schechter A.N., Gladwin M.T. (2002). Cell-free hemoglobin limits nitric oxide bioavailability in sickle-cell disease. Nat. Med..

[58] Rubin O., Crettaz D., Tissot J.-D., Lion N. (2010). Microparticles in stored red blood cells: Submicron clotting bombs?. Blood Transfus..

[59] Zöller B., Li X., Sundquist J., Sundquist K. (2012). Autoimmune diseases and venous thromboembolism: A review of the literature. Am. J. Cardiovasc. Dis..

[60] Esmon C.T. (2005). The interactions between inflammation and coagulation. Br. J. Haematol..

[61] Kamphuisen P.W., Lensen R., Houwing-Duistermaat J.J., Eikenboom J.C.J., Harvey M., Bertina R.M., Rosendaal F.R. (2000). Heritability of elevated factor VIII antigen levels in factor V Leiden families with thrombophilia. Br. J. Haematol..

[62] Lindemann S., Krämer B., Seizer P., Gawaz M. (2007). Platelets, inflammation and atherosclerosis. J. Thromb. Haemost..

[63] Medcalf R.L. (2007). Fibrinolysis, inflammation, and regulation of the plasminogen activating system. J. Thromb. Haemost..

[64] Zwaal R.F.A., Schroit A.J. (1997). Pathophysiologic Implications of membrane phospholipid asymmetry in blood cells. Blood.

[65] Ungprasert P., Tanratana P., Srivali N. (2015). Autoimmune hemolytic anemia and venous thromboembolism: A systematic review and meta-analysis. Thromb. Res..

[66] Schreiber K., Sciascia S., De Groot P.G., Devreese K., Jacobsen S., Ruiz-Irastorza G., Salmon J.E., Shoenfeld Y., Shovman O., Hunt B.J. (2018). Antiphospholipid syndrome. Nat. Rev. Dis. Prim..

[67] Rottem M., Krause I., Fraser A., Stojanovich L., Rovensky J., Shoenfeld Y. (2006). Autoimmune hemolytic anaemia in the antiphospholipid syndrome. Lupus.

[68] Ames P.R., Merashli M., Bucci M.M.T., Pastori D., Pignatelli P., Arcaro A., Gentile F. (2020). Antiphospholipid antibodies and autoimmune haemolytic anaemia: A systematic review and meta-analysis. Int. J. Mol. Sci..

[69] Roumier M., Loustau V., Guillaud C., Languille L., Mahevas M., Khellaf M., Limal N., Noizat-Pirenne F., Godeau B., Michel M. (2014). Characteristics and outcome of warm autoimmune hemolytic anemia in adults: New insights based on a single-center experience with 60 patients. Am. J. Hematol..

[70] Foley J.H. (2016). Examining coagulation-complement crosstalk: Complement activation and thrombosis. Thromb. Res..

[71] Merle N.S., Church S.E., Fremeaux-Bacchi V., Roumenina L.T. (2015). Complement system part I—Molecular mechanisms of activation and regulation. Front. Immunol..

[72] Wiedmer T., Esmon C.T., Sims P.J. (1986). On the mechanism by which complement proteins C5b-9 increase platelet prothrombinase activity. J. Biol. Chem..

[73] Ritis K., Doumas M., Mastellos D., Micheli A., Giaglis S., Magotti P., Rafail S., Kartalis G., Sideras P., Lambris J.D. (2006). A novel C5a receptor-tissue factor cross-talk in neutrophils links innate immunity to coagulation pathways. J. Immunol..

[74] Hattori R., Hamilton K.K., McEver R.P., Sims P.J. (1989). Complement proteins C5b-9 induce secretion of high molecular weight multimers of endothelial von Willebrand factor and translocation of granule membrane protein GMP-140 to the cell surface. J. Biol. Chem..

[75] Foreman K.E., Vaporciyan A.A., Bonish B.K., Jones M.L., Johnson K.J., Glovsky M.M., Eddy S.M., Ward P.A. (1994). C5a-induced expression of P-selectin in endothelial cells. J. Clin. Investig..

[76] Krarup A., Wallis R., Presanis J.S., Gál P., Sim R.B. (2007). Simultaneous activation of complement and coagulation by MBL-associated serine protease 2. PLoS ONE.

[77] Wojta J., Kaun C., Zorn G., Ghannadan M., Hauswirth A.W., Sperr W.R., Fritsch G., Printz D., Binder B.R., Schatzl G. (2002). C5a stimulates production of plasminogen activator inhibitor-1 in human mast cells and basophils. Blood.

[78] Berentsen S. (2015). Role of complement in autoimmune hemolytic anemia. Transfus. Med. Hemother..

[79] Berentsen S. (2018). Complement activation and inhibition in autoimmune hemolytic anemia: Focus on cold agglutinin disease. Semin. Hematol..

[80] Gralnick H.R., Vail M., McKeown L.P., Merryman P., Wilson O., Chu I., Kimball J. (1995). Activated platelets in paroxysmal nocturnal haemoglobinuria. Br. J. Haematol..

[81] Peerschke E.I.B., Andemariam B., Yin W., Bussel J.B. (2010). Complement activation on platelets correlates with a decrease in circulating im-mature platelets in patients with immune thrombocytopenic purpura. Br. J. Haematol..

[82] Devine D.V., Siegel R.S., Rosse W.F. (1987). Interactions of the platelets in paroxysmal nocturnal hemoglobinuria with complement. Relationship to defects in the regulation of complement and to platelet survival in vivo. J. Clin. Investig..

[83] Wiedmer T., Esmon C.T., Sims P.J. (1986). Complement proteins C5b-9 stimulate procoagulant activity through platelet prothrombinase. Blood.

[84] Blair P., Flaumenhaft R. (2009). Platelet α-granules: Basic biology and clinical correlates. Blood Rev..

[85] Hugel B., Socié G., Vu T., Toti F., Gluckman E., Freyssinet J.-M., Scrobohaci M.-L. (1999). Elevated levels of circulating procoagulant microparticles in patients with paroxysmal nocturnal hemoglobinuria and aplastic anemia. Blood.

[86] van Bijnen S.T.A., van Heerde W.L., Muus P. (2012). Mechanisms and clinical implications of thrombosis in paroxysmal nocturnal hemoglobinuria. J. Thromb. Haemost..

[87] Massberg S., Grahl L., Von Bruehl M.-L., Manukyan D., Pfeiler S., Goosmann C., Brinkmann V., Lorenz M., Bidzhekov K., Khandagale A.B. (2010). Reciprocal coupling of coagulation and innate immunity via neutrophil serine proteases. Nat. Med..

[88] Sloand E.M., Pfannes L., Scheinberg P., More K., Wu C.O., Horne M., Young N.S. (2008). Increased soluble urokinase plasminogen activator receptor (suPAR) is associated with thrombosis and inhibition of plasmin generation in paroxysmal nocturnal hemoglobinuria (PNH) patients. Exp. Hematol..

[89] Ninomiya H., Hasegawa Y., Nagasawa T., Abe T. (1997). Excess soluble urokinase-type plasminogen activator receptor in the plasma of patients with paroxysmal nocturnal hemoglobinuria inhibits cell-associated fibrinolytic activity. Int. J. Hematol..

[90] Griffin M., Hillmen P., Munir T., Richards S., Arnold L., Riley K., Hill A. (2019). Significant hemolysis is not required for thrombosis in paroxysmal nocturnal hemoglobinuria. Haematologica.

[91] Connors J.M. (2017). Thrombophilia testing and venous thrombosis. N. Engl. J. Med..

[92] Devreese K.M.J., Ortel T.L., Pengo V., De Laat B. (2018). Laboratory criteria for antiphospholipid syndrome: Communication from the SSC of the ISTH. J. Thromb. Haemost..

[93] Nafa K., Bessler M., Mason P., Vulliamy T., Hillmen P., Castro-Malaspina H., Luzzatto L. (1996). Factor V Leiden mutation investigated by amplification created restriction enzyme site (ACRES) in PNH patients with and without thrombosis. Haematologica.

[94] Grünewald M., Siegemund A., Grünewald A., Schmid A., Koksch M., Schöpflin C., Schauer S., Griesshammer M. (2003). Plasmatic coagulation and fibrinolytic system alterations in PNH: Relation to clone size. Blood Coagul. Fibrinolysis.

[95] Dragoni F., Iori A.P., Pignoloni P., Minotti C., Chiarotti F., Mazzucconi M.G., Mengarelli A., Arcese W., Foà R., Avvisati G. (2010). Thrombophilic screening in patients with paroxysmal nocturnal haemoglobinuria: A pilot study. Br. J. Haematol..

[96] Huang Y., Liu X., Chen F., Zhou W., Li H., Long Z., Yang C., Chen M., Han B. (2019). Prediction of thrombosis risk in patients with paroxysmal nocturnal hemoglobinuria. Ann. Hematol..

[97] Bode C., Olivier C.B., Duerschmied D. (2019). Anticoagulation and anaemia: Old opponents from the era of VKA?. Eur. Heart J..

[98] Stolz E., Valdueza J.M., Grebe M., Schlachetzki F., Schmitt E., Madlener K., Rahimi A., Kempkes-Matthes B., Blaes F., Gerriets T. (2007). Anemia as a risk factor for cerebral venous thrombosis? An old hypothesis revisited: Results of a prospective study. J. Neurol..

[99] Hung S.-H., Lin H.-C., Chung S.-D. (2015). Association between venous thromboembolism and iron-deficiency anemia. Blood Coagul. Fibrinolysis.

[100] Kearon C., Akl E.A., Ornelas J., Blaivas A., Jimenez D., Bounameaux H., Huisman M., King C.S., Morris T.A., Sood N. (2016). Antithrombotic therapy for VTE disease: CHEST guideline and expert panel report. Chest.

[101] Patriquin C.J., Kiss T., Caplan S., Chin-Yee I., Grewal K., Grossman J., Larratt L., Marceau D., Nevill T., Sutherland D.R. (2018). How we treat paroxysmal nocturnal hemoglobinuria: A consensus statement of the Canadian PNH Network and review of the national registry. Eur. J. Haematol..

[102] Emadi A., Brodsky R.A. (2009). Successful discontinuation of anticoagulation following eculizumab administration in paroxysmal nocturnal hemoglobinuria. Am. J. Hematol..

[103] Jäger U., Barcellini W., Broome C.M., Gertz M.A., Hill A., Hill Q.A., Jilma B., Kuter D.J., Michel M., Montillo M. (2020). Diagnosis and treatment of autoimmune hemolytic anemia in adults: Recommendations from the First International Consensus Meeting. Blood Rev..

[104] Hill Q.A., Stamps R., Massey E., Grainger J.D., Provan D., Hill A. (2016). The diagnosis and management of primary autoimmune haemolytic anaemia. Br. J. Haematol..

[105] Weinkle T.K., Center S.A., Randolph J.F., Warner K.L., Barr S.C., Erb H.N. (2005). Evaluation of prognostic factors, survival rates, and treatment protocols for immune-mediated hemolytic anemia in dogs: 151 cases (1993–2002). J. Am. Vet. Med. Assoc..

[106] Moyo V.M., Mukhina G.L., Garrett E.S., Brodsky R.A. (2004). Natural history of paroxysmal nocturnal haemoglobinuria using modern diag-nostic assays. Br. J. Haematol..

[107] Schrezenmeier H., Muus P., Socié G., Szer J., Urbano-Ispizua A., Maciejewski J.P., Brodsky R.A., Bessler M., Kanakura Y., Rosse W. (2014). Baseline characteristics and disease burden in patients in the International Paroxysmal Nocturnal Hemoglobinuria Registry. Haematologica.

[108] Thomsen R.W., Schoonen W.M., Farkas D.K., Riis A., Fryzek J.P., Sørensen H.T. (2010). Risk of venous thromboembolism in splenectomized patients compared with the general population and appendectomized patients: A 10-year nationwide cohort study. J. Thromb. Haemost..

[109] Stuck A., Spirk D., Schaudt J., Kucher N. (2017). Risk assessment models for venous thromboembolism in acutely ill medical patients: A systematic review. J. Vasc. Surg. Venous Lymphat. Disord..

[110] Van’t Riet M., Burger J.W.A., Van Muiswinkel J.M., Kazemier G., Schipperus M.R., Bonjer H.J. (2000). Diagnosis and treatment of portal vein thrombosis following splenectomy. Br. J. Surg..

[111] Mohren M., Markmann I., Dworschak U., Franke A., Maas C., Mewes S., Jentsch-Ullrich K. (2004). Thromboembolic complications after splenectomy for hematologic diseases. Am. J. Hematol..

[112] Kelly R.J., Höchsmann B., Szer J., Kulasekararaj A., De Guibert S., Röth A., Weitz I.C., Armstrong E., Risitano A.M., Patriquin C.J. (2015). Eculizumab in pregnant patients with paroxysmal nocturnal hemoglobinuria. N. Engl. J. Med..

